# Aquaglyceroporins in Human Breast Cancer

**DOI:** 10.3390/cells12172185

**Published:** 2023-08-31

**Authors:** Teresa Kirkegaard, Andreas Riishede, Trine Tramm, Lene N. Nejsum

**Affiliations:** 1 Department of Clinical Medicine, Aarhus University, 8200 Aarhus N, Denmark; au634207@uni.au.dk (T.K.); and@clin.au.dk (A.R.); tramm@clin.au.dk (T.T.); 2 Department of Molecular Biology and Genetics, Aarhus University, 8000 Aarhus C, Denmark; 3 Department of Pathology, Aarhus University Hospital, 8200 Aarhus N, Denmark

**Keywords:** AQP, aquaporin, aquaglyceroporin, breast cancer, metastasis

## Abstract

Aquaporins are water channels that facilitate passive water transport across cellular membranes following an osmotic gradient and are essential in the regulation of body water homeostasis. Several aquaporins are overexpressed in breast cancer, and AQP1, AQP3 and AQP5 have been linked to spread to lymph nodes and poor prognosis. The subgroup aquaglyceroporins also facilitate the transport of glycerol and are thus involved in cellular metabolism. Transcriptomic analysis revealed that the three aquaglyceroporins, AQP3, AQP7 and AQP9, but not AQP10, are overexpressed in human breast cancer. It is, however, unknown if they are all expressed in the same cells or have a heterogeneous expression pattern. To investigate this, we employed immunohistochemical analysis of serial sections from human invasive ductal and lobular breast cancers. We found that AQP3, AQP7 and AQP9 are homogeneously expressed in almost all cells in both premalignant in situ lesions and invasive lesions. Thus, potential intervention strategies targeting cellular metabolism via the aquaglyceroporins should consider all three expressed aquaglyceroporins, namely AQP3, AQP7 and AQP9.

## 1. Introduction

Aquaporins (AQPs) are transmembrane proteins that facilitate transport of water across cellular membranes following an osmotic gradient. There are 13 mammalian AQPs, which are expressed in a multitude of different tissues and cell types, and they are involved in the regulation of water reabsorption and secretion. AQPs are homotetramers, where each monomer forms a pore, allowing bidirectional water transport by diffusion following the osmotic gradient. In addition to water, some AQPs also transport other substances like glycerol and urea (aquaglyceroporins) and are thus involved in energy metabolism (for recent review [1]). The aquaglyceroporins comprise AQP3, AQP7, AQP9 and AQP10; of note, in contrast to the other aquaglyceroporins, AQP10 facilitated glycerol transport may be gated by pH [2,3]. Moreover, some AQPs transport H_2_O_2_ (peroxiporins) and are thus involved in redox signaling and the cellular response to oxidative stress. The peroxiporins comprise AQP1 [4,5], AQP3 [6], AQP5 [7], AQP6 [8], AQP8 [9,10], AQP9 [11] and AQP11 [12]. In addition, *AQP7* knockdown in breast cancer cells resulted in decreased oxidative stress tolerance [13].

AQPs are essential for the regulation of body water homeostasis, and thus, in a wide range of both acquired and congenital diseases associated with dysregulation of water balance, expression and/or subcellular localization of one or more AQPs are found to be altered. For example, this is observed in nephrogenic diabetes insipidus [14], congestive heart failure [15,16] and lithium treatment [17], which are associated with dysregulation of AQP2 in renal collecting duct principal cells. 

Of note, several AQPs have been reported to be overexpressed in cancers like pancreatic cancer and female breast cancers (for reviews [18,19,20,21,22,23]), and AQPs are involved in multiple cellular processes like cellular proliferation, adhesion, migration and polarity as well as the secretion of matrix metalloproteinases (MMPs); which are processes involved in cancer development and spread. The following are select examples of these regulatory processes. For a comprehensive, recent review regarding AQP functions we refer to [1]. AQPs have been implicated in cellular proliferation in multiple cell lines. For example, deletion of *Aqp3* in mice reduced proliferation of keratinocytes [24] and deletion of *Aqp7* in mice reduced proliferation of pancreatic β cells [25]. AQPs also regulate both cell–cell adhesion and cell-extracellular matrix (ECM) adhesion. In normal epithelial Madin-Darby canine kidney (MDCK) cells, AQP3 overexpression increased levels of junctional proteins at cell–cell junctions, whereas AQP1, AQP4 and AQP5 expression decreased levels [26]. Of note, while the effect of AQP7 and AQP9 on epithelial cell–cell adhesion is still not clear, a recent structural study suggested that AQP7 may function as a cell–cell adhesion protein [27]. For a review regarding the role of AQPs in cell–cell adhesion, we refer to [28]. Several AQPs have also been shown to be involved in cellular migration. AQPs localized at the leading edge of migrating cells induce a slight swelling, allowing increased space for actin polymerization and, hence, forward movement (reviewed in [29,30]). Interestingly, in MDCK cells, the overexpression of AQP3, AQP4 and AQP5 all decreased collective cell migration [26], but in contrast, decreased AQP5, via knockdown, decreased the collective migration of MCF7 breast cancer cells [31], and AQP5 overexpression in MCF7 cells increased cellular migration [32], indicating that the effect may be cell-type-specific. Interestingly, AQP5 overexpression in the normal MDCK cell line induced cell detachment from the front of migrating cell sheets, which was mediated by induction of Ras signaling via the serine 156 in the second intracellular loop of AQP5 [26]. Neither AQP1, AQP3, nor AQP4 induced cell detachment from migrating MDCK cell sheets [26]. In cultured glioblastoma cells, AQP1 induced the upregulation of the Focal Adhesion Kinase (FAK) [33] and AQP4 expression in D54 glioma cells increased cell adhesion to the ECM [34]. AQP expression also affects the secretion of MMPs, which degrade the extracellular matrix. In human gastric cancer cell lines, AQP3 induced expression of MT1-MMP, MMP2 and MMP9 [35]. Overexpression of AQP5 in MDCK cells caused disruption of 3D grown spheroids, and of note, localization of AQP5 and the major polarity protein Scribble seemed inversely correlated in human breast cancer samples [32]. AQPs also interact with multiple proteins, which may be important for their subcellular localization and function (reviewed in [36]). In pull-down assays, AQP5 interacted with Scribble and several proteins involved in cell–cell adhesion [26,32,37], which may be important for the effect of AQP5 on cellular junctions and cellular polarity. It is still not known if these interactions are direct or indirect. 

Female breast cancer is the most frequently diagnosed cancer, with a yearly incidence in 2020 of 2.3 million and 685,000 deaths [38]. The vast majority of breast cancers arise in the terminal duct lobular unit (TDLU) [39,40], and carcinomas arising from the epithelial cells of the mammary glands are the most common type of breast cancer. Carcinoma in situ is a pre-invasive diagnosis characterized by dysplastic cells confined to the epithelium and surrounded by a layer of myoepithelial cells and an intact basement membrane. Carcinoma in situ lesions are not cancers since they do not invade the surrounding tissue and do not have the potential to metastasize. Subclassifications of carcinoma in situ are ductal carcinoma in situ (DCIS) (80%) and lobular carcinoma in situ (LCIS) (20%) [39], where especially DCIS (and subtypes of LCIS) are considered non-obligate pre-cursor lesions with varying risk of progression to cancer. In invasive carcinomas, malignant cells have invaded the surrounding stroma and thus migrated through the layer of myoepithelial cells and breached the basement membrane. Invasive carcinomas are classified based on tissue morphology, with the most common subtypes being invasive ductal carcinoma (IDC) (60–75%) and invasive lobular carcinoma (ILC) (10–15%) [39,40]. 

Transcriptomic analyses using the Human Protein Atlas database revealed higher mRNA levels of several AQPs in human breast cancer, namely *AQP1*, *AQP3*, *AQP5*, *AQP7*, *AQP9* and *AQP11*, when compared to overall median AQP expression levels in the biopsies [41]. High protein expression of AQP1, AQP3 (triple-negative breast cancer) and AQP5 (early and triple-negative breast cancer) revealed a correlation with spread to lymph nodes and poor prognosis [18,31,42,43,44]. Moreover, a study found that breast cancer patients with high *AQP7* mRNA expression had reduced overall survival (OS) compared to patients with low *AQP7* mRNA expression [13] and high *AQP9* mRNA expression was associated with a worse relapse-free survival (RFS) and OS [45]. Therefore, the AQPs have been suggested as prognostic markers and potential intervention targets. 

To obtain a comprehensive insight into how AQPs affect breast cancer development, spread and invasion, it is essential to know if the AQPs are expressed and localized in the same regions and cells. However, the transcriptomic analysis [41] did not provide this information and immunohistochemical analyses are most often not performed on serial sections or with double labeling, thus not revealing if the AQPs are expressed/localized in the same regions and cells. Thus, with a focus on the aquaglyceroporins AQP3, AQP7 and AQP9, which have been shown to be expressed in breast cancer and affect spread and/or survival, we aimed to investigate protein localization in the same regions and cells in human, female breast cancer tumors using immunohistochemistry on serial sections. 

## 2. Materials and Methods

### 2.1. Patient Samples

Formalin-fixed, paraffin-embedded samples from invasive female breast cancer tumors were obtained in fully anonymized form from 18 surgical specimens at the Department of Pathology at Aarhus University Hospital, Denmark. The study was in compliance with the *Danish Act on Processing of Personal Data and Health*, and since the material encompassed only fully anonymized tissue that was not used for diagnostic purposes and otherwise subjected to discard, informed consent from the patients was not required (Komitéloven § 14 Stk.3). Of the 18 samples, 10 were previously characterized and described [32]. Since the material was fully anonymized, no clinicopathological data were available and thus not included. Histological type (invasive ductal or lobular carcinoma, etc.) was determined using bright-field microscopy based on Hematoxylin–Eosin (HE) stained slides supplemented with immunohistochemical stainings for E-cadherin. The E-cadherin immunohistochemical staining was performed with a rabbit anti-E-cadherin antibody diluted 1:100 (Santa Cruz Biotechnology cat# SC8426, Dallas, TX, USA) following the protocol described below with the secondary antibody goat-anti-mouse diluted 1:500 (Agilent Technologies, cat# P0447, Santa Clara, CA, USA). Only samples of invasive ductal (11 samples) and invasive lobular (5 samples) carcinoma were included. Thus, two samples were excluded (1 was metaplastic carcinoma, and 1 was DCIS with only small areas of invasive carcinoma). To test the distribution of the aquaglyceroporins AQP3, AQP7 and AQP9 in the same tumor regions and cells, immunohistochemistry on serial sections was performed, followed by a qualitative evaluation. 

### 2.2. Immunohistochemistry

Immunohistochemistry was performed as previously described [32,46]. Paraffin embedded tissue was sectioned on a microtome into 2 μm thick serial sections and placed on SuperFrost^®^ Plus glass slides. The sections were dried on a heating block for 20 min at 60 °C and deparaffinized by overnight incubation in xylene. Rehydration was performed by incubation in 99% ethanol (2 × 10 min) followed by 96% ethanol (2 × 10 min). Endogenous peroxidase was blocked by incubation for 30 min in 30% H_2_O_2_ dissolved in methanol. Next, the sections were incubated in 70% ethanol (10 min) and rinsed 3–4× in distilled water. The sections were subjected to epitope retrieval by heat in a rice cooker for 20 min in TEG buffer (10 mM Tris, 0.5 mM EGTA, pH 9) and subsequently placed on ice for 30 min. Aldehyde groups were shielded by incubation for 30 min in shielding buffer (50 mM NH_4_Cl in PBS). The slides were then placed horizontally, and a line was drawn around the tissue sections with a PAP pen and sections were blocked by 3 × 10 min incubations in blocking buffer (1% BSA, 0.2% gelatin, 0.05% saponin in PBS). Next, sections were incubated overnight at 4°C in a humidity chamber with primary antibodies diluted in staining buffer (0.1% BSA, 0.3% Triton X-100 in PBS). The previously validated primary antibodies were: section #1 rabbit anti-AQP7 diluted 1:200 [47], section #2 rabbit-anti-AQP9 diluted 1:50 [48,49], section #3 rabbit anti-AQP3 diluted 1:400 (Alomone Labs, Cat# AQP-003, Jerusalem, Israel) and section #4 mouse-anti-Smooth Muscle Myosin, heavy chain (SMMS-1) diluted 1:2000 (Cell Marque, Cat# 298M-15, Rocklin, CA, USA). Section #4, SMMS-1, was used to label myoepithelial cells and thus enable differentiation of in situ lesions from invasive lesions. 

The next day, the sections were equilibrated to room temperature and subsequently washed 3 × 10 min in washing buffer (0.1% BSA, 0.2% gelatin, 0.05% saponin in PBS) and incubated for 1 h with goat-anti-rabbit antibodies diluted 1:500 (Agilent Technologies, cat# P0448) or goat-anti-mouse antibodies diluted 1:500 (Agilent Technologies, cat# P0447) in staining buffer followed by 3 × 10 min wash in washing buffer. Next, sections were incubated with 3,3′-diaminobenzidine (DAB) (Agilent Technologies, cat# K3468) for 10 min, followed by washing in PBS (3 × 10 min) and 1x in demineralized water. The sections were counterstained with Mayer’s hematoxylin (3 min) and washed for 20 min in tap water. Lastly, the sections were dehydrated (1 × 5 min in 70% ethanol, 2 × 5 min in 96% ethanol and 2 × 5 min in 99% ethanol) and mounted with coverslips using Eukitt mounting medium (Sigma Aldrich, cat# 03989, St. Louis, MO, USA). Controls included omission of primary antibodies.

### 2.3. Microscopy and Image Analysis

The stained serial sections were imaged using a bright-field Leica DMLB microscope equipped with a DeltaPix INVENIO 6EIII color camera with 5× (NA 0.11) and 40× (NA 0.65) air objectives. Several images were captured for each slide. For publication quality figures, select slides were scanned in a NanoZoomer 2.0HT (Hamamatsu, Shizuoka, Japan) using the 40× objective. Cropped regions were generated in QuPath-0.4.3 [50] and exported to ImageJ Fiji 1.53v for analysis [36]. The same regions in the serial sections were identified and a crop generated whereafter white balance was adjusted in Adobe Photoshop CS4 to best present tissue morphology via the hematoxylin counterstain. The images were manually evaluated, and the regions were manually aligned to present the same regions for the four stainings.

## 3. Results 

### 3.1. AQP3, AQP7 and AQP9 Are Expressed in Epithelial Cells of Normal Lobules and Extralobular Ducts in Benign Structures Adjacent to Tumor Tissue

**Figure 1:**
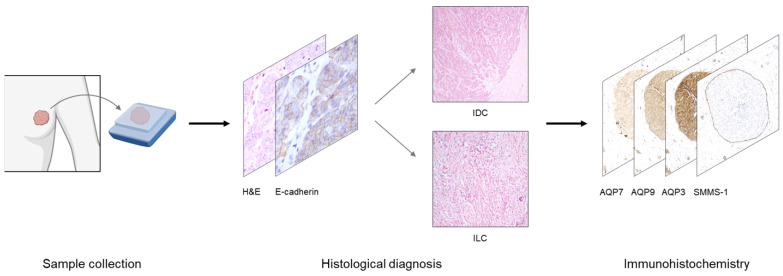
Graphical illustration of workflow. Formalin-fixed, paraffin-embedded samples from invasive female breast cancer tumors were obtained in fully anonymized form from surgical specimens from the Department of Pathology, Aarhus University Hospital, Denmark. Following retrieval, the anonymized tissue samples were sectioned. For diagnostic assessment, hematoxylin and eosin (H&E) stainings, as well as immunohistochemical stainings for E-cadherin, were performed. Samples representing invasive ductal carcinoma (IDC) and invasive lobular carcinoma (ILC) were further analyzed. Serial sections were processed for immunohistochemical stainings in the following order: #1 AQP7, #2 AQP9, #3 AQP3 and #4 SMMS-1. The SMMS-1 staining identified myoepithelial cells, allowing a distinction between in situ and invasion lesions. Images of all samples were captured by brightfield light microscopy, and select slides for publication figures were scanned on a NanoZoomer 2.0HT (Hamamatsu) with the 40× objective. Part of this figure was generated in BioRender.com; accessed on August 11th 2023.

To first establish the distribution of AQP3, AQP7 and AQP9 in mammary epithelium (experimental setup in Figure 1), we investigated acini in lobules and extralobular ducts in benign breast structures adjacent to tumor tissue in the immunohistochemical stainings of serial sections with antibodies against AQP3, AQP7 and AQP9, as well as the marker for myoepithelial cells (SMMS-1). 

**Figure 2:**
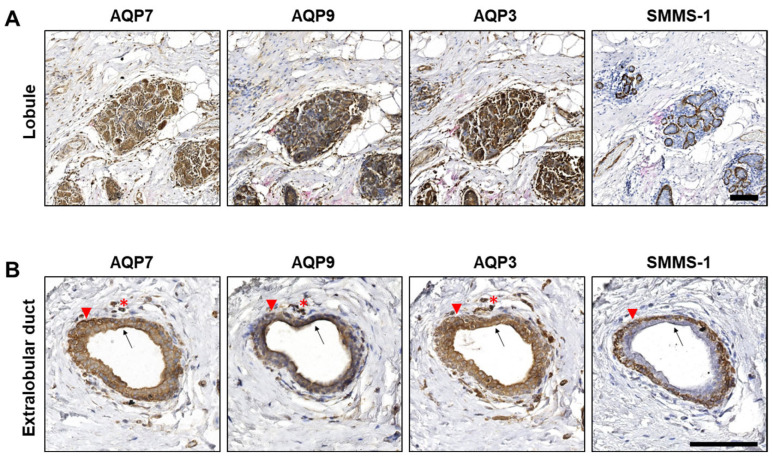
AQP7, AQP9 and AQP3 localize to epithelial cells of lobules and extralobular ducts in benign regions adjacent to tumor tissue. Formalin-fixed, paraffin-embedded samples from invasive female breast cancer tumors were obtained in fully anonymized form from surgical specimens and processed for immunohistochemistry with antibodies against AQP7, AQP9, AQP3 and SMMS-1. The figure depicts representative images from immunohistochemical stainings of serial sections from the normal/benign part of samples from invasive lobular carcinoma. (**A**) Lobule and (**B**) extralobular duct. Immune cells in the surrounding connective tissue also stain positive (red asterisks in B). Arrowheads in B point to myoepithelial cells, and the arrows in B point to the apical region of extralobular ducts. Slides were scanned in a NanoZoomer 2.0HT (Hamamatsu) using the 40× objective. White balance was adjusted in Adobe Photoshop CS4. Scale bars are 100 µm.

The immunohistochemical stainings revealed that the epithelial cells of the acini in the lobules (Figure 2A) and in the extralobular ducts (Figure 2B) were positive for all three aquaglyceroporins. AQP3, AQP7 and AQP9 localized both intracellularly and in the plasma membrane. Apical accentuation was observed in ducts (arrows), but not in acini. As expected, immunohistochemical staining for the marker for myoepithelial cells (SMMS-1) was observed in myoepithelial cells surrounding lobules and extralobular ducts (Figure 2A,B). Interestingly, in extralobular ducts (Figure 2B), AQP3, AQP7 and AQP9 also localized to the myoepithelial cells (arrowheads).

We thus found that AQP3, AQP7 and AQP9 are expressed in all mammary epithelial cells in both lobules and extralobular ducts but that the subcellular distribution varies. It is important to emphasize that the present samples were collected to encompass malignant tumors. The breast glands in the samples are thus lying in proximity to the malignant lesions, and it cannot be excluded that the expression and distribution of AQP7, AQP9 and AQP3 are affected within close areas surrounding the lesions. However, our data indicate that not only AQP3, as previously shown [37], but also AQP7 and AQP9 are expressed in normal human mammary glands where they may contribute to water and glycerol transport and, thus, cellular metabolism. 

### 3.2. AQP3, AQP7 and AQP9 Are Homogeneously Distributed in Both Premalignant In Situ Regions and in Invasive Breast Cancer

**Figure 3:**
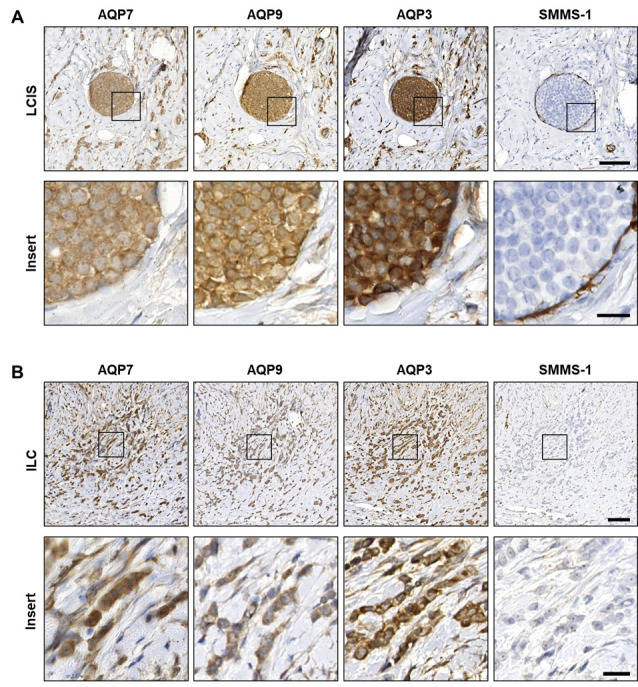
AQP7, AQP9 and AQP3 localize to neoplastic cells of lobular carcinoma in situ and invasive lobular carcinoma. Formalin-fixed, paraffin-embedded samples from invasive female breast cancer tumors were obtained in fully anonymized form from surgical specimens and processed for immunohistochemistry with antibodies against AQP7, AQP9, AQP3 and SMMS-1. The figure depicts representative images from immunohistochemical stainings of serial sections from patients with invasive lobular carcinoma (ILC) and enlarged images (inserts) of the marked areas. (**A**) Images are from a region of lobular carcinoma in situ (LCIS) from an ILC patient, and (**B**) is ILC. Slides were scanned in a NanoZoomer 2.0HT (Hamamatsu) using the 40× objective. White balance was adjusted in Adobe Photoshop CS4. Scale bars are 100 µm (**A**,**B**) and 20 µm ((**A**,**B**), inserts).

**Figure 4:**
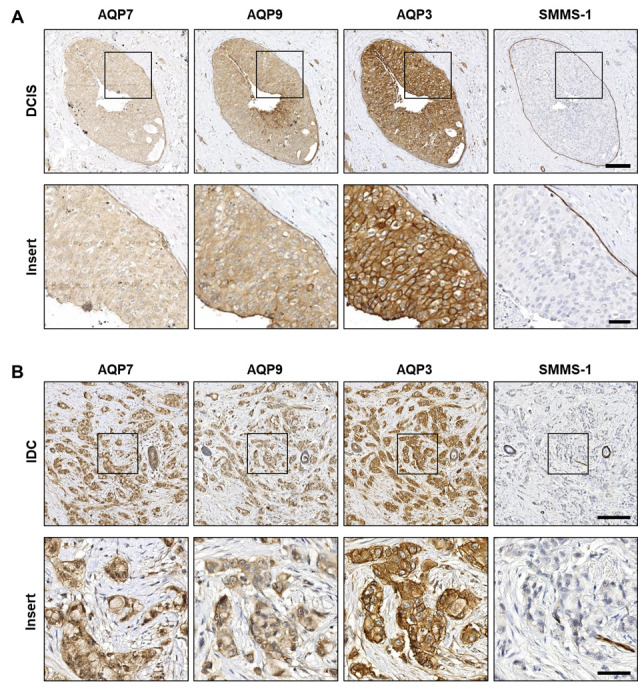
AQP7, AQP9 and AQP3 localize to neoplastic cells of ductal carcinoma in situ and invasive ductal carcinoma. Formalin-fixed, paraffin-embedded samples from invasive female breast cancer tumors were obtained in fully anonymized form from surgical specimens and processed for immunohistochemistry with antibodies against AQP7, AQP9, AQP3 and SMMS-1. The figure depicts representative images from immunohistochemical stainings of serial sections from patients with invasive ductal carcinoma (IDC) and enlarged images (inserts) of the marked areas. (**A**) Images are from a region of ductal carcinoma in situ (DCIS) from an IDC patient, and (**B**) is IDC. Slides were scanned in a NanoZoomer 2.0HT (Hamamatsu) using the 40x objective. White balance was adjusted in Adobe Photoshop CS4. Scale bars are 175 µm (**A**,**B**) and 50 µm ((**A**,**B**), inserts).

All tumor samples were obtained from invasive cancers; however, immunohistochemical stainings with the myoepithelial marker SMMS-1 revealed several regions with in situ lesions where the myoepithelial cell layer surrounding the malignant cells was intact (Figure 3A and Figure 4A). Positive AQP3, AQP7 and AQP9 immunohistochemical stainings were observed in all the premalignant LCIS and DCIS regions (representative examples in Figure 3A and Figure 4A, respectively), as well as in the invasive ILC and IDC regions (representative examples in Figure 3B and Figure 4B, respectively). Localization of AQP3, AQP7 and AQP9 in LCIS was clearly observed in the plasma membrane, although intracellular localization could also be observed (Figure 3A). In ILC, AQP7 localization was observed both in plasma membranes and intracellularly, whereas localization of AQP3 and AQP9 seemed mainly to be in the plasma membrane (Figure 3B). 

In the premalignant DCIS regions (Figure 4A), both plasma membrane localization as well as intracellular localization could be observed. Plasma membrane staining was especially prominent for AQP3. In IDC (Figure 4B), immunohistochemical stainings revealed that AQP3 predominantly localized to the plasma membrane while AQP7 and AQP9 predominantly localized intracellularly. 

In general, the immunohistochemical staining patterns observed in the individual tumor islands were mostly homogeneous, although heterogeneity sometimes occurred, with some tumor cells having increased positive staining compared to the adjacent tumor cells within the same tumor island. Staining intensities seemed to vary across a sample, and this effect seemed identical for the three aquaglyceroporins. This could be due to intratumor heterogeneity [51] but we cannot exclude that it is an artifact that originates from pre-analytical factors, including immersion fixation.

## 4. Discussion

Several AQPs have been detected in human mammary glands. AQP3 was expressed in both ducts and glands [52] and moreover, in benign structures adjacent to breast cancer tumor tissue, AQP1 was expressed in myoepithelial cells [43] and AQP5 in ductal epithelial cells [31]. By analyzing the immunohistochemical stainings of AQP3, AQP7 and AQP9 in the normal/benign part of the samples, we confirmed the expression of AQP3 in epithelial cells of both lobules and extralobular ducts and also detected AQP7 and AQP9, indicating that AQP7 and AQP9 may also be involved in mammary function and fluid secretion. 

A previous study performing transcriptomic analyses on breast cancer biopsies revealed increased mRNA expression of *AQP1*, *AQP3*, *AQP5*, *AQP7*, *AQP9* and *AQP11* when compared to overall median AQP levels in the biopsies [41]. Therefore, to further characterize aquaglyceroporin expression, the AQP3, AQP7 and AQP9 protein distribution pattern was investigated in the serial sections. The fourth aquaglyceroporin, AQP10, was omitted from this analysis since transcriptomic analysis indicated that AQP10 expression was unchanged in breast cancer biopsies. AQP3, AQP7 and AQP9 were very homogenous, with the vast majority of cells staining positive. This contrasts the expression of AQP5, where the staining pattern in breast cancer samples was heterogeneous [32]. The homogenous expression of AQP3, AQP7 and AQP9 suggests that the cancer cells have a high capacity for glycerol uptake, which may contribute to the increased energy consumption of the cancer cells. Indeed, metabolomics and lipid profiling of breast cancer cells revealed that AQP7 was involved in lipid metabolism, glutathione metabolism, and urea/arginine metabolism [13]. In addition to glycerol transport, several AQPs also facilitate H_2_O_2_ transport, which includes the two aquaglyceroporins AQP3 [6] and AQP9 [11]. *AQP7* knockdown in breast cancer cells resulted in decreased oxidative stress tolerance [13]. Thus, the homogenous expression of AQP3, AQP7 and AQP9 may aid the cancer cells in their adaptations to high osmotic stress as well as facilitate H_2_O_2_-mediated signaling—both of which may aid cancer cell survival. 

AQP expression may also facilitate cancer spread via increased secretion of MMPs that degrade the basement membrane, as well as facilitate increased cancer cell migration by degrading the extracellular matrix. Several AQPs have been shown to increase MMP secretion in various cell types. AQP3 overexpression increased MMP-3 secretion in prostate cancer cells [53] and MT1-MMP, MMP-2, and MMP-9 in gastric cancer cells [35]. Moreover, *AQP9* knockdown in human chondrocytes decreased mRNA levels of MMP3 MMP13 as well as a disintegrin and metalloproteinase with thrombospondin motifs 5 (ADAMTS-5) [54]. Thus, the combination of AQP3 and AQP9 expression may contribute to increased MMP secretion in breast cancer. It is still unknown if AQP7 facilitates increased MMP secretion, and it is unknown how the combinatory effects of the three AQPs affect MMP secretion in breast cancer cells. 

Of note, we recently found that AQP1, AQP3 and AQP5 differentially affected the response of 3D-grown human breast cancer spheroids to conventional anticancer therapies [55]. AQP3 overexpression reduced cell viability in response to Cisplatin, 5-Fluorouracil (5-FU) and Doxorubicin, as well as a combination of the three treatments. Viability in AQP1-overexpressing spheroids was decreased by all treatments except for 5-FU, whereas only Doxorubicin and the combination decreased viability of AQP5 overexpressing spheroids. Systematic analysis of how AQP7 and AQP9 affect the response of breast cancer cells to conventional anticancer therapies is still lacking. Moreover, future analysis should focus both on individual AQPs as well as combinations and how the expression affects the response to anticancer therapies. 

Targeting the aquaglyceroporins as well as the peroxiporins holds the potential to impair cancer cell metabolism and response to oxidative stress. However, since all three aquaglyceroporins are expressed in the same cells, and several peroxiporins are expressed in breast cancer (AQP1 [4,5], AQP3 [6] AQP5 [7] and AQP9 [11] as well as AQP7 (*AQP7* knockdown in breast cancer cells resulted in decreased oxidative stress tolerance [13])), targeted intervention strategies of only one AQP may encounter compensatory mechanisms. Therefore, a strategy targeting multiple AQPs with overlapping functions may be more beneficial. 

## 5. Conclusions

In conclusion, by using immunohistochemical analysis of serial sections from human breast cancer samples from invasive lobular and ductal carcinoma, we found that the three aquaglyceroporins—AQP3, AQP7 and AQP9—are homogeneously expressed in the samples from invasive ductal and lobular cancers as well as the regions of ductal and lobular carcinoma in situ. While the expression of several AQPs in breast cancer may affect multiple cellular features that facilitate increased invasion and spread and worsen prognosis, the unique, combined AQP expression also holds the potential for the design of personalized anticancer therapies that target AQP expression and/or function or sensitizes tumors to anticancer therapies.

## Data Availability

Data and materials used in the analysis can be made available in some form upon reasonable request to any researcher for purposes of reproducing or extending the analysis.

## References

[1] Login F.H., Nejsum L.N. (2023). Aquaporin water channels: Roles beyond renal water handling. Nat. Rev. Nephrol..

[2] Zhang L., Yao D., Xia Y., Zhou F., Zhang Q., Wang Q., Qin A., Zhao J., Li D., Li Y. (2021). The structural basis for glycerol permeation by human AQP7. Sci. Bull..

[3] Gotfryd K., Mosca A.F., Missel J.W., Truelsen S.F., Wang K., Spulber M., Krabbe S., Helix-Nielsen C., Laforenza U., Soveral G. (2018). Human adipose glycerol flux is regulated by a pH gate in AQP10. Nat. Commun..

[4] Montiel V., Bella R., Michel L.Y.M., Esfahani H., De Mulder D., Robinson E.L., Deglasse J.P., Tiburcy M., Chow P.H., Jonas J.C. (2020). Inhibition of aquaporin-1 prevents myocardial remodeling by blocking the transmembrane transport of hydrogen peroxide. Sci. Transl. Med..

[5] Wang H., Schoebel S., Schmitz F., Dong H., Hedfalk K. (2020). Characterization of aquaporin-driven hydrogen peroxide transport. Biochim. Biophys. Acta Biomembr..

[6] Miller E.W., Dickinson B.C., Chang C.J. (2010). Aquaporin-3 mediates hydrogen peroxide uptake to regulate downstream intracellular signaling. Proc. Natl. Acad. Sci. USA.

[7] Rodrigues C., Pimpao C., Mosca A.F., Coxixo A.S., Lopes D., da Silva I.V., Pedersen P.A., Antunes F., Soveral G. (2019). Human Aquaporin-5 Facilitates Hydrogen Peroxide Permeation Affecting Adaption to Oxidative Stress and Cancer Cell Migration. Cancers.

[8] Pellavio G., Martinotti S., Patrone M., Ranzato E., Laforenza U. (2022). Aquaporin-6 May Increase the Resistance to Oxidative Stress of Malignant Pleural Mesothelioma Cells. Cells.

[9] Bienert G.P., Moller A.L., Kristiansen K.A., Schulz A., Moller I.M., Schjoerring J.K., Jahn T.P. (2007). Specific aquaporins facilitate the diffusion of hydrogen peroxide across membranes. J. Biol. Chem..

[10] Bertolotti M., Bestetti S., Garcia-Manteiga J.M., Medrano-Fernandez I., Dal Mas A., Malosio M.L., Sitia R. (2013). Tyrosine kinase signal modulation: A matter of H_2_O_2_ membrane permeability?. Antioxid. Redox Signal..

[11] Watanabe S., Moniaga C.S., Nielsen S., Hara-Chikuma M. (2016). Aquaporin-9 facilitates membrane transport of hydrogen peroxide in mammalian cells. Biochem. Biophys. Res. Commun..

[12] Bestetti S., Galli M., Sorrentino I., Pinton P., Rimessi A., Sitia R., Medrano-Fernandez I. (2020). Human aquaporin-11 guarantees efficient transport of H_2_O_2_ across the endoplasmic reticulum membrane. Redox Biol..

[13] Dai C., Charlestin V., Wang M., Walker Z.T., Miranda-Vergara M.C., Facchine B.A., Wu J., Kaliney W.J., Dovichi N.J., Li J. (2020). Aquaporin-7 Regulates the Response to Cellular Stress in Breast Cancer. Cancer Res..

[14] Deen P.M., Verdijk M.A., Knoers N.V., Wieringa B., Monnens L.A., van-Os C.H., van-Oost B.A. (1994). Requirement of human renal water channel aquaporin-2 for vasopressin-dependent concentration of urine. Science.

[15] Nielsen S., Terris J., Andersen D., Ecelbarger C., Frokiaer J., Jonassen T., Marples D., Knepper M.A., Petersen J.S. (1997). Congestive heart failure in rats is associated with increased expression and targeting of aquaporin-2 water channel in collecting duct. Proc. Natl. Acad. Sci. USA.

[16] Xu D.L., Martin P.Y., Ohara M., St John J., Pattison T., Meng X., Morris K., Kim J.K., Schrier R.W. (1997). Upregulation of aquaporin-2 water channel expression in chronic heart failure rat. J. Clin. Investig..

[17] Marples D., Christensen S., Christensen E.I., Ottosen P.D., Nielsen S. (1995). Lithium-induced downregulation of aquaporin-2 water channel expression in rat kidney medulla. J. Clin. Investig..

[18] Traberg-Nyborg L., Login F.H., Edamana S., Tramm T., Borgquist S., Nejsum L.N. (2022). Aquaporin-1 in breast cancer. APMIS.

[19] Bystrup M., Login F.H., Edamana S., Borgquist S., Tramm T., Kwon T.H., Nejsum L.N. (2022). Aquaporin-5 in breast cancer. APMIS.

[20] Bruun-Sorensen A.S., Edamana S., Login F.H., Borgquist S., Nejsum L.N. (2021). Aquaporins in pancreatic ductal adenocarcinoma. APMIS.

[21] Tomita Y., Dorward H., Yool A.J., Smith E., Townsend A.R., Price T.J., Hardingham J.E. (2017). Role of Aquaporin 1 Signalling in Cancer Development and Progression. Int. J. Mol. Sci..

[22] Marlar S., Jensen H.H., Login F.H., Nejsum L.N. (2017). Aquaporin-3 in Cancer. Int. J. Mol. Sci..

[23] Jensen H.H., Login F.H., Koffman J.S., Kwon T.H., Nejsum L.N. (2016). The role of aquaporin-5 in cancer cell migration: A potential active participant. Int. J. Biochem. Cell Biol..

[24] Hara-Chikuma M., Verkman A.S. (2008). Prevention of skin tumorigenesis and impairment of epidermal cell proliferation by targeted aquaporin-3 gene disruption. Mol. Cell. Biol..

[25] Matsumura K., Chang B.H., Fujimiya M., Chen W., Kulkarni R.N., Eguchi Y., Kimura H., Kojima H., Chan L. (2007). Aquaporin 7 is a beta-cell protein and regulator of intraislet glycerol content and glycerol kinase activity, beta-cell mass, and insulin production and secretion. Mol. Cell. Biol..

[26] Login F.H., Jensen H.H., Pedersen G.A., Koffman J.S., Kwon T.H., Parsons M., Nejsum L.N. (2019). Aquaporins differentially regulate cell-cell adhesion in MDCK cells. FASEB J..

[27] Huang P., Venskutonyte R., Prasad R.B., Ardalani H., de Mare S.W., Fan X., Li P., Spegel P., Yan N., Gourdon P. (2023). Cryo-EM structure supports a role of AQP7 as a junction protein. Nat. Commun..

[28] Edamana S., Login F.H., Yamada S., Kwon T.H., Nejsum L.N. (2021). Aquaporin water channels as regulators of cell-cell adhesion proteins. Am. J. Physiol. Cell Physiol..

[29] Papadopoulos M.C., Saadoun S., Verkman A.S. (2008). Aquaporins and cell migration. Pflugers Arch..

[30] Smith I.M., Stroka K.M. (2023). The multifaceted role of aquaporins in physiological cell migration. Am. J. Physiol. Cell Physiol..

[31] Jung H.J., Park J.Y., Jeon H.S., Kwon T.H. (2011). Aquaporin-5: A marker protein for proliferation and migration of human breast cancer cells. PLoS ONE.

[32] Edamana S., Login F.H., Riishede A., Dam V.S., Tramm T., Nejsum L.N. (2023). The cell polarity protein Scribble is downregulated by the water channel aquaporin-5 in breast cancer cells. Am. J. Physiol. Cell Physiol..

[33] Oishi M., Munesue S., Harashima A., Nakada M., Yamamoto Y., Hayashi Y. (2020). Aquaporin 1 elicits cell motility and coordinates vascular bed formation by downregulating thrombospondin type-1 domain-containing 7A in glioblastoma. Cancer Med..

[34] McCoy E., Sontheimer H. (2007). Expression and function of water channels (aquaporins) in migrating malignant astrocytes. Glia.

[35] Xu H., Xu Y., Zhang W., Shen L., Yang L., Xu Z. (2011). Aquaporin-3 positively regulates matrix metalloproteinases via PI3K/AKT signal pathway in human gastric carcinoma SGC7901 cells. J. Exp. Clin. Cancer Res..

[36] Roche J.V., Tornroth-Horsefield S. (2017). Aquaporin Protein-Protein Interactions. Int. J. Mol. Sci..

[37] Login F.H., Palmfeldt J., Cheah J.S., Yamada S., Nejsum L.N. (2021). Aquaporin-5 regulation of cell-cell adhesion proteins: An elusive “tail” story. Am. J. Physiol. Cell Physiol..

[38] Sung H., Ferlay J., Siegel R.L., Laversanne M., Soerjomataram I., Jemal A., Bray F. (2021). Global Cancer Statistics 2020: GLOBOCAN Estimates of Incidence and Mortality Worldwide for 36 Cancers in 185 Countries. CA Cancer J. Clin..

[39] Nolan E., Lindeman G.J., Visvader J.E. (2023). Deciphering breast cancer: From biology to the clinic. Cell.

[40] Makki J. (2015). Diversity of Breast Carcinoma: Histological Subtypes and Clinical Relevance. Clin. Med. Insights Pathol..

[41] Chow P.H., Bowen J., Yool A.J. (2020). Combined Systematic Review and Transcriptomic Analyses of Mammalian Aquaporin Classes 1 to 10 as Biomarkers and Prognostic Indicators in Diverse Cancers. Cancers.

[42] Lee S.J., Chae Y.S., Kim J.G., Kim W.W., Jung J.H., Park H.Y., Jeong J.Y., Park J.Y., Jung H.J., Kwon T.H. (2014). AQP5 expression predicts survival in patients with early breast cancer. Ann. Surg. Oncol..

[43] Qin F., Zhang H., Shao Y., Liu X., Yang L., Huang Y., Fu L., Gu F., Ma Y. (2016). Expression of aquaporin1, a water channel protein, in cytoplasm is negatively correlated with prognosis of breast cancer patients. Oncotarget.

[44] Zhu Z., Jiao L., Li T., Wang H., Wei W., Qian H. (2018). Expression of AQP3 and AQP5 as a prognostic marker in triple-negative breast cancer. Oncol. Lett..

[45] Zhu L., Ma N., Wang B., Wang L., Zhou C., Yan Y., He J., Ren Y. (2019). Significant prognostic values of aquaporin mRNA expression in breast cancer. Cancer Manag. Res..

[46] Jensen H.H., Login F.H., Park J.Y., Kwon T.H., Nejsum L.N. (2017). Immunohistochemical evalulation of activated Ras and Rac1 as potential downstream effectors of aquaporin-5 in breast cancer in vivo. Biochem. Biophys. Res. Commun..

[47] Lebeck J., Ostergard T., Rojek A., Fuchtbauer E.M., Lund S., Nielsen S., Praetorius J. (2012). Gender-specific effect of physical training on AQP7 protein expression in human adipose tissue. Acta Diabetol..

[48] Lindskog C., Asplund A., Catrina A., Nielsen S., Rutzler M. (2016). A Systematic Characterization of Aquaporin-9 Expression in Human Normal and Pathological Tissues. J. Histochem. Cytochem..

[49] Elkjaer M., Vajda Z., Nejsum L.N., Kwon T., Jensen U.B., Amiry-Moghaddam M., Frokiaer J., Nielsen S. (2000). Immunolocalization of AQP9 in liver, epididymis, testis, spleen, and brain. Biochem. Biophys. Res. Commun..

[50] Bankhead P., Loughrey M.B., Fernandez J.A., Dombrowski Y., McArt D.G., Dunne P.D., McQuaid S., Gray R.T., Murray L.J., Coleman H.G. (2017). QuPath: Open source software for digital pathology image analysis. Sci. Rep..

[51] Polyak K. (2011). Heterogeneity in breast cancer. J. Clin. Investig..

[52] Mobasheri A., Wray S., Marples D. (2005). Distribution of AQP2 and AQP3 water channels in human tissue microarrays. J. Mol. Histol..

[53] Chen J., Wang Z., Xu D., Liu Y., Gao Y. (2015). Aquaporin 3 promotes prostate cancer cell motility and invasion via extracellular signal-regulated kinase 1/2-mediated matrix metalloproteinase-3 secretion. Mol. Med. Rep..

[54] Takeuchi K., Hayashi S., Matumoto T., Hashimoto S., Takayama K., Chinzei N., Kihara S., Haneda M., Kirizuki S., Kuroda Y. (2018). Downregulation of aquaporin 9 decreases catabolic factor expression through nuclear factor-kappaB signaling in chondrocytes. Int. J. Mol. Med..

[55] Edamana S., Pedersen S.F., Nejsum L.N. (2023). Aquaporin water channels affect the response of conventional anticancer therapies of 3D grown breast cancer cells. Biochem. Biophys. Res. Commun..

